# Prevalence, characteristics, and impacts of work-related musculoskeletal disorders: a survey among physical therapists in the State of Kuwait

**DOI:** 10.1186/1471-2474-11-116

**Published:** 2010-06-11

**Authors:** Hesham N Alrowayeh, Talal A Alshatti, Sameera H Aljadi, Majda Fares, Mishayek M Alshamire, Sahar S Alwazan

**Affiliations:** 1 Kuwait University, Faculty of Allied Health Sciences, Physical Therapy Department, Kuwait City, State of Kuwait; 2 Ministry of Health, Kuwait City, State of Kuwait

## Abstract

**Background:**

Physical therapists working in the State of Kuwait are at risk of work-related musculoskeletal disorders (WMSDs). However, prevalence rates and risk factors are not well documented. The objective of this study was to determine the prevalence, characteristics, and impacts of WMSDs among physical therapists in the State of Kuwait.

**Methods:**

A self-administered questionnaire was distributed to 350 physical therapists. The questionnaire gathered demographic data as well as information on occurrence of musculoskeletal complaints in the previous 12 months. Descriptive statistics, frequency, and Chi-square analyses were used.

**Results:**

The response rate to the questionnaire was 63% (222/350). Of the 212 responders included in the study, the one-year prevalence of WMSDs was 47.6%, with lower back complaints as the most common (32%). This was followed by neck (21%), upper back (19%), shoulder (13%), hand/wrist (11%), knee (11%), ankle/foot (6%), elbow (4%), and hip/thigh (3%) complaints. The frequency of WMSDs was not gender related (except lower back, neck, and shoulder complaints) nor was it related to age (except lower back complaints), working venues (except hand/wrist), working hours, area of specialty, or exercise. WMSDs' impact on work was minor.

**Conclusions:**

WMSDs among physical therapists in Kuwait were common, with lower back and neck affected most. Lower back and neck WMSDs were related to the participant's demographics. Hand/wrist WMSDs were related to work settings. Further research is needed to investigate the effect of risk factors as physical load, psychosocial load, and general health status on prevalence musculoskeletal disorders.

## Background

Work-related musculoskeletal disorders (WMSDs) are injuries that result from work events [1]. Physical therapists are often at risk for developing WMSDs because they are often involved in physically demanding and intense, repetitive tasks in their practices [2,3].The highest prevalence of WMSDs among physical therapists is reported to be in the lower back area [1-11].

There is little information about the prevalence of WMSDs among physical therapists and other health care professionals in Kuwait. Two recent studies reported the prevalence of lower back complaints [10,12]. They did not, however, report on other areas of the body. Shehab et al. demonstrated high lifetime prevalence (70%) of lower back pain among physical therapists due to improper body mechanics and faulty technique during the daily activities of patient handling [10]. Landry et al. investigated the prevalence and factors associated with low back pain (LBP) among health care providers in an urban orthopedic hospital in Kuwait [12]. The lifetime prevalence of LBP in their study was 70%. The factors associated with the LBP in their sample were the number of daily lifts and transfers performed by the health professionals [12]. These findings contradict the findings from other studies conducted worldwide [1,4,5,11].

Prevalence of neck, shoulder, elbow, hand/wrist, upper back, hip/thigh, knee, and ankle/foot WMSDs among physical therapists in the State of Kuwait remain unknown. Therefore, the objective of this study was to determine the prevalence, characteristics, and impacts of WMSDs in all anatomical areas of the body among physical therapists in the State of Kuwait. The findings from the study could help identify overall WMSDs among physical therapists and eventually contribute to the development of prevention and intervention strategies.

## Methods

### Participants

Physical therapists of all nationalities working in public or private practice in various specialties (e.g., neurology, cardiology, burn, orthopedic, geriatric, pediatric, etc.) in the State of Kuwait participated in this study. Only physical therapists with at least one year of work experience in their current work settings were non-randomly selected to participate in this study. All participants read and signed the informed consent approved by the Ethical Review Board of Kuwait University.

### Instrument

A three-part, self-administered questionnaire was used in this study (Additional file 1). Part one collected the participant's personal characteristics and included questions about age, gender, family history, and exercise habits. Part two collected information on the participant's education and current work history. It included questions regarding level of education, duration of employment, working settings, and professional rank. Part three assessed occurrence of musculoskeletal complaints using a standardized Nordic questionnaire. This questionnaire divides the human body into nine anatomical regions (neck, shoulder, elbow, hand/wrist, upper back, lower back, hip/thigh, knee, and ankle/foot) on the basis of two criteria: regions where symptoms tend to accumulate and regions that are distinguishable from each other [13]. The questionnaire included a diagram with the anatomical regions clearly marked. Participants are asked whether they have or have had troubles in the indicated areas during the preceding 12 months [13].

### Procedures

Three hundred and fifty copies of the questionnaire were distributed among prospective participants who were recruited by convenience sampling. A trained physical therapists distributed the questionnaire. The physical therapists explained the questionnaire to each participant and provided a contact number in case further explanation would be required. The completed copies of the questionnaire were collected by the same physical therapist within one week. The study procedures was approved by the Ethical Review Board of Kuwait University.

### Data analyses

Descriptive statistics were used to estimate the prevalence of WMSDs and demographic characteristics. Frequencies and cross-tabulations were used to compare musculoskeletal disorders prevalence between demographics (gender, age, etc.) and work history (experience,setting, specialty, etc.). Chi-square tests were also used to assess these relationships.Statistical significance was evaluated at α = 0.05.

## Results

Of the three hundred fifty physical therapists, 222 (63%) physical therapists completed/returned the questionnaires. Ten questionnaires were excluded from analysis because participants had less than one year of experience in their current work settings. Thus, only data from 212 participants were used to calculate the prevalence rates. No one question was missing more than 5% of the responses. The time taken to complete the questionnaire was 10 to 15 minutes.

### Participants description

There were more male than female physical therapists who participated in this study (Table 1); the mean age was 36.5 ± 9.1 years (range = 22-63 years). Most participants (80%) had lesser than 20 years of clinical experience, mean 14.0 ± 5.3 years (Table 1). The majority of participants worked in government hospitals/schools (Table 1). Orthopedics was the most common area of specialty (Table 1). Seventy-one percent worked 40 hours a week or more (Table 1). Twenty-six percent of participants were ranked as physical therapist practitioners, and the majority of participants held entry level degrees (84%) (Table 1).

**Table 1:** Demographic description of study participants

Gender	**No**.	%
Male	113	53
Female	99	47

**Age**		

20-30	45	21
31-40	112	53
41-50	27	13
50 +	28	13

**Nationality**		

Kuwaiti	74	35
Egyptian	29	14
Indian	70	33
Other	48	18

**Education**		

BSc	178	84
MSc	19	9
PhD	5	2

**Professional Rank**		

Junior PT	38	18
PT	55	26
Senior PT	40	19
PT specialist	30	15
Senior PT specialist	26	13
Superintendent PT	17	9

**Professional experience**		

0-10	91	43
11-20	79	37
20+	42	20

**Working Hours**		

< 10	12	6
10-19	4	2
20-29	19	10
30-39	19	10
40 +	150	71

**Working Venues**		

General hospital	75	35
Private hospital/clinic	8	4
Rehabilitation hospital	84	40
Specialized hospital	38	18
School	4	2

**Areas of Specialty**		

Neurology	44	21
Cardiology	16	8
Burns/plastic	1	0.5
Orthopedics	59	28
Geriatrics	1	0.5
Pediatrics	30	14
Sports Medicine	6	3
Others	26	12

Column values do not always total overall sample due to missing data

Others nationalities include: Saudi, Romanian, Bulgarian, Iraqi, Lebanese, Palestinian

Others areas of specialty include: general practice, maternity, and special needs school

### Prevalence rate of WMSDs

The one-year prevalence of WMSDs among physical therapists in the study was 47.6%. Lower back complaint was the most common WMSDs (Table 2). The prevalence of work-related lower back complaints was significantly associated with the participant's gender (*p *= .005), with more female than male reporting lower back complaints (Table 2). Similarly, the prevalence of work-related lower back complaints was significantly associated with participants age (*p *= .01) and occurred more often in the younger age groups (20-40 years) (Table 2). However, the prevalence of lower back WMSDs was not significantly associated with working venue, areas of specialty, working hours, or exercise habits (Table 2).

**Table 2:** Prevalence and association of work-related musculoskeletal disorders among physical therapists working in Kuwait to demographics, work settings, and exercise habits.

	Neck			Shoulder			Elbow			Hand/Wrist			Upper back			Lower back			Hip/thigh			Knee			Ankle/feet		
	**No**.	**%**	***p***	**No**.	**%**	***p***	**No**.	**%**	***p***	**No**.	**%**	***p***	**No**.	**%**	***p***	**No**.	**%**	***p***	**No**.	**%**	***p***	**No**.	**%**	***p***	**No**.	**%**	***p***
**Gender**																											

**Male**	5	2.3		2	0.9		2	0.9		3	1.4		8	4.0		20	9.4		0	0		7	3.3		3	1.4	
**Female**	38	17.9	.0005	25	11.7	.001*	6	2.8	.13	20	9.4	.21	32	15.0	.37	48	22.6	.005*	7	3.3	.08	16	7.5	.79	10	4.7	.52

**Age**																											

**20-30**	13	6.1		9	4.2		4	1.8		8	3.7		14	6.6		26	12.2		2	0.9		10	4.7		5	2.3	
**31-40**	26	12.2		15	7.0		3	1.4		13	6.1		22	10.3		31	14.6		4	1.8		6	2.8		5	2.3	
**41-50**	4	1.8		2	0.9		0	0		2	0.9		3	1.4		8	3.7		1	0.4		4	1.8		3	1.0	
**50 +**	0	0	.16	2	0.9	.85	1	0.4	.63	0	0	.21	1	0.4	.38	3	1.4	.01*	0	0	.92	3	1.4	.39	0	0	.33

**Working venues**																											

**General hosp**	21	9.9		6	2.8		4	1.8		7	3.3		14	6.6		28	13.2		4	1.8		7	3.3		6	2.8	
**Private hosp**	1	0.4		0	0		0	0		1	0.4		1	0.4		3	1.4		0	0		0	0		1	0.4	
**Rehabil hosp**	16	7.5		13	6.1		3	1.4		13	6.1		15	7.0		22	10.3		1	0.4		11	5.1		4	1.8	
**Specialized hosp**	4	1.8		6	2.8		1	0.4		1	0.4		9	4.2		11	5.1		2	0.9		3	1.4		2	0.9	
**School**	1	0.4	.68	1	0.4	.32	0	0	.49	0	0	.01*	0	0	.44	2	0.9	.91	0	0	.23	1	0.4	.46	0	0	.64

**Area of Specialty**																											

**Neurology**	6	2.8		4	1.8		0	0		4	1.8		4	1.8		10	4.7		1	0.4		1	0.4		3	1.4	
**Cardiology**	2	0.9		2	0.9		0	0		2	0.9		3	1.4		5	2.3		1	0.4		2	0.9		1	0.4	
**Burns**	0	0		1	0.4		0	0		0	0		1	0.4		1	0.4		0	0		0	0		0	0	
**Orthopedics**	12	5.6		7	3.3		4	1.8		3	1.4		10	4.7		21	9.9		2	0.9		6	2.8		2	0.9	
**Geriatrics**	0	0		0	0		0	0		0	0		1	0.4		1	0.4		0	0		1	0.4		1	0.4	
**Pediatrics**	11	5.1		5	2.3		0	0		7	3.3		8	3.7		14	6.6		1	0.4		9	4.2		1	0.4	
**Sports**	2	0.9		1	0.4		2	0.9		1	0.4		2	0.9		2	0.9		0	0		0	0		2	0.9	
**Other**	1	0.4	.70	2	0.9	.5	2	0.9	> .05	0	0	.19	5	2.3	.89	5	2.3	.85	2	0.9	.69	1	0.4	.1	0	0	.75

**Working Hours**																											

**< 10**	1	0.4		2	0.9		1	0.4		3	1.4		4	1.8		4	1.8		0	0		1	0.4		1	0.4	
**10-19**	0	0		0	0		0	0		0	0		0	0		0	0		1	0.4		0	0		0	0	
**20-29**	3	1.4		2	0.9		0	0		1	0.4		2	0.9		4	1.8		0	0		3	1.4		0	0	
**30-39**	10	4.7		5	2.3		2	0.9		1	0.4		8	3.7		15	7		2	0.9		7	3.3		3	1.4	
**40 +**	27	12.7	.91	16	7.5	.7	5	2.3	.75	16	7.5	.43	23	10.8	.84	44	20.7	.71	4	1.8	.34	12	5.6	.69	9	4.2	.77

**Exercise**																											

**Yes**	27	12.7		16	7.5		5	2.3		12	5.6		26	12.2		38	17.9		4	1.8		10	4.7		6	2.8	
**No**	17	8.02	.66	11	5.1	049	3	1.4	.43	11	5.1	.13	14	6.6	.76	30	14.1	.39	3	1.4	.97	13	6.1	.07	7	3.3	.55

Other areas of specialty include: general practice, maternity, and special needs school

* Indicates significant association at α = 0.05.

Neck complaint was the second most prevalent WMSDs (Table 2). The prevalence of neck WMSDs was significantly associated with gender (*p *= .0005), occurring more often among the female participants (Table 2). However, age, working venues, areas of specialty, working hours, and exercise habits were not significantly associated with the prevalence of neck WMSDs (Table 2).

Prevalence of upper back (19%), shoulder (13%), hand/wrist (11%), knee (11%), ankle/foot (6%), elbow (4%), and hip/thigh (3%) complaints followed lower back and neck WMSDs (Table 2). The prevalence of the upper back, shoulder, hand/wrist, knee, ankle/foot, elbow, and hip/thigh WMSDs were not significantly associated with participant's gender (except shoulder, *p *= .001) and age, work venues (except hand/wrist, *p *= .01), areas of specialty, working hours, or exercise habits (Table 2).

### Characteristics of WMSDs

The majority of participating physical therapists had one to five episodes of neck, shoulder, elbow, hand/wrist, upper back, lower back, hip/thigh, knee, and ankle/foot WMSDs (Table 3). The majority of WMSD episodes lasted 1-7 days (except elbow), for a total of fewer than 28 days (Table 3). Pain was the most common complaint. This was followed by cramp/spasm and stiffness, and other symptoms varied (Table 3). Symptoms developed gradually in most participants (Table 3).

**Table 3:** Characteristics of WMSDs among physical therapists working in Kuwait

	Neck		Shoulder		Elbow		Hand/Wrist		Upper back		Lower back		Hip/thigh		Knee		Ankle/feet	
	
	**No**.	%	**No**.	%	**No**.	%	**No**.	%	**No**.	%	**No**.	%	**No**.	%	**No**.	%	**No**.	%
Longest spell (days)																		
1 to 7	59	65.6	21	48.8	2	18.2	15	55.5	32	59.3	51	52.6	7	50.0	25	67.6	13	54.2
8 to 21	17	18.9	7	16.3	7	63.6	2	7.4	8	14.8	18	18.6	4	28.6	8	21.6	3	12.5
22 to 30	4	4.4	2	4.7	0	0	3	11.1	2	3.7	8	8.2	0	0	1	2.7	4	16.7
31 to 93	2	2.2	4	9.3	0	0	5	18.5	3	5.6	5	5.2	1	7.1	2	5.4	3	12.5
94+	8	8.9	9	20.9	2	18.2	2	7.4	3	16.7	15	15.5	2	14.3	1	2.7	1	4.2

**Total length of spell (days)**																		

< 28	61	67.8	22	51.2	7	63.6	14	53.8	34	64.2	57	58.8	9	64.3	27	73.0	14	58.3
29 to 93	12	13.3	9	20.9	1	9.1	9	34.6	5	9.4	18	18.6	2	14.3	9	24.3	5	20.8
94 to 66	10	11.1	5	11.6	1	9.1	0	0	4	7.5	8	8.2	0	0	1	2.7	4	16.7
67+	8	7.8	7	16.3	2	18.2	3	11.5	10	18.9	14	14.1	3	21.4	0	0	1	4.2

**No. of spell**																		

1	22	25.3	6	14.6	4	40	7	25.0	8	15.4	16	16.8	4	30.8	3	13.1	5	20.8
2 to 5	47	54.0	20	48.8	2	20.0	10	35.7	29	55.8	51	53.7	4	30.8	16	69.5	15	62.5
5+	18	20.7	15	36.6	4	40.0	11	39.3	15	28.8	28	29.5	5	38.5	4	17.4	4	16.7

**Nature of complaints**																		

Stiffness	44	50.0	15	34.9	2	18.2	5	17.9	25	47.2	52	53.6	2	14.3	14	37.8	3	12.5
Nagging feeling	10	11.4	11	25.6	3	27.3	5	17.9	13	24.5	19	19.6	4	28.6	4	10.8	4	16.7
Numbness	6	6.8	4	9.3	1	9.1	12	42.9	4	7.5	14	14.4	3	21.4	1	2.7	2	8.3
Tingling	6	6.8	2	4.7	2	18.2	8	28.6	5	9.4	5	5.2	1	7.1	3	8.1	1	4.2
Loss of strength	8	9.1	11	25.6	3	27.3	9	32.1	7	13.2	21	21.6	2	14.3	10	27.0	0	0
Cramp, spasm	67	76.1	23	53.5	4	36.4	6	21.4	41	77.4	65	67.0	8	57.1	7	18.9	5	20.8
Pain	69	78.4	39	90.7	11	100.0	24	85.7	48	90.6	89	91.8	12	85.7	36	97.3	24	100.0

**Treatment received**																		

No Rx	12	14.0	10	24.2	5	50.0	3	11.5	11	22.4	13	13.4	5	35.7	15	41.7	10	43.5
PT	40	46.5	18	43.9	1	10.0	14	53.8	23	46.9	41	42.3	5	35.7	12	33.3	7	30.4
Drugs	13	15.1	8	19.5	1	10.0	5	19.2	6	12.2	10	10.3	2	14.3	5	13.9	1	4.3
PT and Drugs	21	24.4	5	12.2	3	30.0	4	15.4	9	18.4	32	33	2	14.3	4	11.1	4	7.4

**Onset of complaints**																		

Sudden	39	44.3	12	27.9	5	45.5	11	39.3	15	28.8	31	33.7	5	35.7	11	30.6	13	54.2
Gradual	49	55.7	31	72.1	6	54.5	17	60.7	37	71.2	61	66.3	9	64.3	25	69.4	11	45.8

**Expert seen**																		

General practitioner	8	9.0	5	11.9	1	9.1	3	10.7	2	3.9	6	6.7	0	0	3	8.3	0	0
Physical therapist	21	23.6	8	19.0	2	18.2	5	17.9	12	23.5	18	20.0	1	8.3	3	8.3	3	13.0
A specialist	15	16.9	3	7.1	0	0	7	25.0	4	7.8	18	20.0	2	16.7	5	13.9	5	21.7

The majority of participants who had neck, shoulder, hand/wrist, upper back, and lower back complaints received physical therapy while those with elbow, knee, and ankle/foot complaints received no treatment (Table 3). Participants with neck, shoulder, elbow, hand/wrist, and upper back WMSDs were generally seen by a physical therapist (Table 3). On the other hand, those with hip/thigh, knee, and ankle/foot WMSDs were usually seen by a specialist (Table 3). Participants with lower back complaints were seen by both a physical therapist and a specialist (Table 3).

### Impact of WMSDs on participants' work

The majority of participating physical therapists made no changes in their work habits, area of practice, nor limited their patient contact as a result of their WMSDs. Most did not take sick leave. However, if sick leave was used, the work days missed ranged from one to seven (Table 4).

**Table 4:** Impact of WMSDs among physical therapists working in Kuwait.

	Neck		Shoulder		Elbow		Hand/Wrist		Upper back		Lower back		Hip/thigh		Knee		Ankle/feet	
	
No. of sick leave taken	**No**.	%	**No**.	%	**No**.	%	**No**.	%	**No**.	%	**No**.	%	**No**.	%	**No**.	%	**No**.	%
0	75	85.2	39	90.7	11	100.0	25	89.3	47	88.7	72	74.2	11	78.6	33	89.2	21	87.5
1	8	9.1	3	7.0	0	0	2	7.1	3	5.7	9	9.3	0	0	2	5.4	0	0
2 to 5	4	4.5	1	2.3	0	0	1	3.6	3	5.7	14	14.4	2	14.3	1	2.7	2	8.3
5 +	1	1.1	0	0	0	0	0	0	0	0	2	2.1	1	7.1	1	2.7	1	4.2
																		

**Total no. of days with sick leave**																		

0	79	89.9	38	90.5	11	100.0	25	89.3	47	88.7	71	73.2	11	78.6	34	91.9	21	87.5
1 to 7	8	9.1	4	9.5	0	0	3	10.7	6	11.3	21	21.6	2	14.3	3	8.1	2	8.3
8 to 14	1	1.1	0	0	0	0	0	0	0	0	3	3.1	0	0	0	0	0	0
15+	0	0	0	0	0	0	0	0	0	0	2	2.1	1	7.1	0	0	2	4.2

## Discussion

### Prevalence rate and characteristics of WMSDs

WMSDs were common among physical therapists working in the State of Kuwait. However, the one-year prevalence rate of WMSDs reported by participants in our study was lower than most rates reported by physical therapists around the world [2,3,9]. We found that 47.6% of the participating physical therapists complained of WMSDs in at least one anatomical area. This was less than the prevalence reported in the United States (61%) [2], Australia (91%) [3], and Nigeria (91.3%) [9]. A possible explanation for this lower rate may be practice differences, with more physical therapist aides being available in the State of Kuwait to help with the varied work-related tasks (e.g., lifting, transferring). Previous researches have linked manual therapy to WMSDs among health professional [2,3,11]. Because manual therapy techniques are not widely or commonly practiced in Kuwait, this could be another possible explanation for the lower rate of WMSDs.

Our finding of higher lower back WMSD prevalence, followed by neck, upper back, shoulders, and hand/wrist complaints, is consistent with previous research [3,7]. These findings correlate WMSDs to the particular therapeutic tasks performed daily by physical therapists (e.g., lifting, transferring, manual therapy) [2,3,6]. These tasks can put stresses on specific anatomical areas (e.g., lower back, neck, upper back, hand/wrist).

The one-year prevalence of lower back WMSDs in this study was 32%. This was less than the reported prevalence among physical therapists in the United States (45%) [2], Nigeria (69.8%) [9], the United Kingdom (37.2%) [7], and Australia (62.5%) [3]. Similarly, we found that the one-year prevalence for the neck, upper back, shoulder, hand/wrist, knee, ankle/foot, elbow, and hip/thigh complaints in this study was lower than reports from the United States, Australia, Nigeria, and the United Kingdom [2,3,7,9].

### Demographics, work settings, and exercise habits

There was no significant association between the participant's age and gender and the prevalence of elbow, upper back, hip/thigh, knee, and ankle/foot WMSDs in this study. However, the prevalence of lower back, neck, and shoulder WMSDs was associated with the participant's gender. In this study, we observed a significantly higher prevalence of lower back complaints among female (22.9%) than male (9.4%) physical therapists. Similarly, 17% and 12% of the female in our study reported neck and shoulder complaints, respectively, whereas 2.3% and 0.9% of the male reported these complaints. These findings were consistent with previous studies [2,7,9,10]. Gender is considered a potential risk factor to develop WMSDs, since female tend to have a smaller body build [2]. In our study, the female participants had smaller body mass indexes (BMI = 26.4 kg/m ^2^) than the male (BMI = 27.3 kg/m ^2^). This is a disadvantage when handling and treating large patients. Pregnancy also appears to have contributed to the higher prevalence of lower back complaints. In this study, 39% of the female participants reported that their lower back complaints were associated with pregnancy.

The prevalence of lower back WMSDs was significantly associated with participant age. In this study, more than 26% of the participants between the ages of 20-40 years had lower back complaints, while only 5% of those older than 40 years did. This association between age and lower back complaints has also been reported in previous studies [2-4,14,15]. The high prevalence of lower back complaints among younger physical therapists could be associated with a lack of professional experience, knowledge and skills [1,4]. Higher work load could be another explanation for the higher prevalence of lower back complaints in younger physical therapists. Moving out of direct patient care and into administrative positions, which are less physically demanding, could explain the lower prevalence of lower back complaints in our study's older age groups.

Except for hand/wrist complaints, the prevalence of WMSDs was not significantly associated with work settings (e.g., work venue, work hours, area of specialty). The increased prevalence of hand/wrist complaints among participants who work in rehabilitation hospitals may be explained by the frequently practiced manual therapy techniques. This finding supports the study of Bork and colleagues [2]. Bork et al. reported that physical therapists who routinely perform manual therapy were 3.5 times more likely to have wrist or hand symptoms than those who did not perform these techniques [2].

The prevalence of WMSDs was not significantly associated with exercise habits. While physical therapists who reported that they exercise showed a higher rate of WMSD, it was not significant. These findings were not expected since exercise and physical activity promote and maintain health and fitness and prevent injury. Landry et al. found, however, that physical therapists in Kuwait with LBP tend to exercise less than those with no back problems [12].

### WMSDs' impact on work

In general, WMSDs' impact on work was minor. No more than 25% of the physical therapists took sick leave because of WMSDs. Physical therapists in this study reported no changes in their work habits, patient contact, or areas of practice. These findings were not consistent with previous research [3,4]. Molumphy et al. found that 41% of physical therapists with work-related lower back complaints took sick leave [4]. Gromie et al. reported that one in six therapists changed settings or left the profession due to WMSDs [3]. The reason for these differences may be cultural, as most physical therapists in Kuwait choose to work for the government. This tends to be a more restrictive setting in terms of changing work habits or areas of practice or limiting patient contact. Another explanation might be the policies and protective measures followed by the physical therapists when lifting/handling patients and using equipments. This might reduce the risk of injury rate, as reported [16,17].

In this study, the majority of participants with WMSDs were seen by a physical therapists. There is evidence that physical therapists tend to treat themselves or seek informal treatment from their colleagues [7]. This is consistent with our findings that more than 30% of the participants had physical therapy as their preferred treatment. This might also explain the lower number of missed work days.

## Limitations

The main limitation of this study was the sampling technique (convenience sampling). This may have affected the generalizability of the findings. The study also relied on self-reported data, and participants may not have recalled all incidents of WMSDs. Further, participants may have underestimated their injuries in order to avoid being stereotyped by their superiors or being viewed negatively based on their history of injuries, which may affect promotion or future of employment opportunities. The study did not investigate the inter-relationship between musculoskeletal disorders and risk factors such as physical load, psychosocial load, and general health status. This, however, is the topic of ongoing research.

## Conclusion

There were three key findings in our study: 1) one-year prevalence of WMSDs among physical therapists in the State of Kuwait was common, with the lower back affected most. This was followed by neck, upper back, shoulder, hand/wrist, knee, ankle/foot, elbow, and hip/thigh areas; 2) WMSDs were not related to the participant's demographics (except neck, shoulder, and lower back), work settings (except hand/wrist), and exercise habits; and 3) work duties were not affected by musculoskeletal disorders. These findings underline the importance of having WMSDs education and prevention programs in the State of Kuwait. The availability of and early access to treatment and prevention programs may prove affective in reducing these injuries.

## Competing interests

We declare that we have no competing interests. Also, in the past five years we have not received reimbursements, fees, funding, or salary from an organization that may in any way gain or lose financially from the publication of this manuscript, either now or in the future. We do not hold any stocks or shares in an organization that may in any way gain or lose financially from the publication of this manuscript, either now or in the future. Currently, we are not applying for any patents relating to the content of the manuscript. We have not received reimbursements, fees, funding, or salary from an organization that holds or has applied for patents relating to the content of the manuscript.

## Authors' contributions

All authors contributed to some or all of the following activities: concept and research design, writing, project management, consultation, data collection and analysis.

Specifically, HNA contributed to project management, concept and research design, writing, data collection and analysis, and fund procurement. TAA contributed to project management, concept and research design, consultation (including review of manuscript before submission), and data collection and analysis. SHA contributed to writing and consultation. MF contributed to consultation (including review of manuscript before submission) and interpretation of data. MMA contributed to consultation (including review of manuscript before submission) and data collection and analysis. SSA contributed to consultation (including review of manuscript before submission) and data collection and analysis. All authors read and approved the final manuscript.

## Pre-publication history

The pre-publication history for this paper can be accessed here:

http://www.biomedcentral.com/1471-2474/11/116/prepub

## Supplementary Material

Additional file 1 **Modified standardized Nordic questionnaire**. A three-part, self-administered questionnaire. Click here for file
