# Supplementary material for: Prevalence, characteristics, and impacts of work-related musculoskeletal disorders: a survey among physical therapists in the State of Kuwait
Source: BMC Musculoskelet Disord. 2010 Jun 11;11:116. doi: 10.1186/1471-2474-11-116 (PMC2905326; doi:10.1186/1471-2474-11-116)
Supplement: Additional file 1 — Modified standardized Nordic questionnaire. A three-part, self-administered questionnaire. [file 1471-2474-11-116-S1.DOC]

KUWAIT UNIVERSITY

FACULTY OF ALLIED HEALTH SCIENCES

PHYSICAL THERAPY DEPARTMENT

Prevalence of Musculoskeletal Disorders

among Physical Therapist in the State of Kuwait

The prevalence of musculoskeletal disorders among physical therapist all over the world is well documented. Yet, it is not studied in the State of Kuwait. Surveying the occurrence of musculoskeletal complaints paves the way to prevention and intervention strategies. Therefore, the purpose of this study (questionnaire) is 1) to determine the prevalence of musculoskeletal disorders and 2) to investigate the inter-relationship between musculoskeletal disorders and physical load, psychosocial factors, and general health status in Kuwaiti Physical Therapist.

You are kindly requested to answer the following questions either by filling the blank or by putting a cross in the appropriate box- one cross for each question. You may be in doubt as to how to answer, but please do your best anyway. Please answer every question, even if you have never had trouble in any part of your body.

**Part 1: Personal characteristics**

Date of filling-out the questionnaire ___ / ___ / 200__

day month  year

1. What is your year of birth? 19____

2. What is your nationality? _________________

3. What is your sex?

❒ Male ❒ Female

4. How tall are you?

____cm

5. What is your weight?

____Kg

6. What is your marital situation?

❒ single

❒ married

❒ divorced

❒ widow/widower

7. Do you have children or invalid persons at your home?

❒ Yes ❒ No

**If yes:**

**-**How many children in the age of 0-3 years? ___ persons

**-**How many children in the age of 4-12 years? ___ persons

**-**How many children in the age of 13-21 years? ___ persons

**-**How many children in the age of 22 or over? ___ persons

**-**How many invalid persons? ___ persons

(ie. Elderly, handicapped persons)

8. Do you exercise?

❒ Yes ❒ No

**If yes:**

How many hours a day do you exercise? ______ hours a day

How many days a week do you exercise? ______ days a week

**Part 2: Education and current job**

1. What is the **highest education** that you have **completed** successfully?

❒ BSc degree

❒ MSc degree

❒ PhD degree

2. When did you start this job? 19____

3. What is your current professional rank?

❒ Junior physical therapist practitioner ❒ Physical therapist specialist

( ممارس مبتدىء علاج طبيعي ( ( اختصاصي علاج طبيعي )

❒ Physical therapist practitioner ❒ Senior physical therapist specialist

( اختصاصي اول علاج طبيعي ) ( ممارس علاج طبيعي )

❒ Senior physical therapist practitioner ❒ head of department superintendent

( ممارس اول علاج طبيعي ) ( رئيس اختصاصي العلاج الطبيعي)

4. When did you have this rank? 19____

5. What is your area of specialty?

❒ neurology ❒ geriatrics

❒ cardiology ❒ pediatrics

❒ burns ❒ general practice

❒ orthopedics ❒ others, specify___________

6. What is your working setting?
 ❒ general hospital

❒ private clinic

❒ private hospital

❒ rehabilitation hospital

❒ specialized hospital

7. How many hours a week do you work in this job (*including regular overtime*)?

____hours a week

8. How many days a week do you work in this job?

____days a week

9. Do you supervise people in your daily work? ❒ yes ❒ no

10. In what shift do you work?

❒ only morning shift

❒ only evening shift

❒ only night shift

# Part 3: Physical Risk Factor associated with the working conditions in the current job

# Indicate which activities in your current job you perform seldom, sometimes, often, or always?

|  | **never** | **sometimes** | **often** | **always** |
| --- | --- | --- | --- | --- |
| 1. standing for long periods………………………. | ❒ | ❒ | ❒ | ❒ |
| 2. sitting for long periods…………………………. | ❒ | ❒ | ❒ | ❒ |
| 3. long periods of Video Display Unit work (i.e.  computer)……………………………………………. | ❒ | ❒ | ❒ | ❒ |
| 4. walking for long periods…………………………. | ❒ | ❒ | ❒ | ❒ |
| 5. working prolonged periods squatting/kneeling….. | ❒ | ❒ | ❒ | ❒ |
| 6. working with your hands above shoulder height… | ❒ | ❒ | ❒ | ❒ |
| 7. working with your hands below knee height……. | ❒ | ❒ | ❒ | ❒ |
| 1. reaching far……………………………………… | ❒ | ❒ | ❒ | ❒ |
| 9. lifting or carrying loads (below 5 Kg)…………... | ❒ | ❒ | ❒ | ❒ |
| 10. lifting or carrying loads (over 5 Kg)……………. | ❒ | ❒ | ❒ | ❒ |
| 11. pushing or pulling loads (over 5 Kg)……………. | ❒ | ❒ | ❒ | ❒ |
| 12. slipping or falling during transport of loads……. | ❒ | ❒ | ❒ | ❒ |
| 13. regularly applying force with hands or arms……. | ❒ | ❒ | ❒ | ❒ |
| 14. working with vibrating hand tools (i.e. massage  machine, US)………………………………………. | ❒ | ❒ | ❒ | ❒ |
| 15. driving in vehicles………………………………. | ❒ | ❒ | ❒ | ❒ |
| 16. bending and/or twisting with your upper body  many times per hour……………………………….. | ❒ | ❒ | ❒ | ❒ |
| 17. working in awkward postures…………………… | ❒ | ❒ | ❒ | ❒ |
| 18. working prolonged periods in the same posture… | ❒ | ❒ | ❒ | ❒ |
| 19. repeating the same movement of your arms or  hands many times per minute……………………… | ❒ | ❒ | ❒ | ❒ |

1. Could you indicate at this scale how you perceive your physical load during regular activities at work?

1 2 3 4 5 6 7 8 9 10 11 12 13 14 15 16 17 18 19 20

# E.g. 6 Very, very little 15 Heavy

1. Very little 17 Very big
2. Little 19 Very, very big
3. Big

# Part 4: Psychological Risk Factor associated with your regular work

| **Indicate how true the following statements are for your current job? You may choose between never, sometimes, often or always** |
| --- |

| **Decision authority** | **never** | **sometimes** | **often** | **always** |
| --- | --- | --- | --- | --- |
| 1.Do you have freedom in carrying out your tasks? ………………………………………… | ❒ | ❒ | ❒ | ❒ |
| 2.Do you have influence on the planning of  your tasks?........................................................... | ❒ | ❒ | ❒ | ❒ |
| 3.Can you influence the pace of your work?....... | ❒ | ❒ | ❒ | ❒ |
| 4.Can you decide yourself how you carry out  your tasks?........................................................... | ❒ | ❒ | ❒ | ❒ |
| 5.Can you briefly interrupt your work if  needed?................................................................ | ❒ | ❒ | ❒ | ❒ |
| 6.Can you decide in which order you carry out  your tasks?............................................................ | ❒ | ❒ | ❒ | ❒ |
| 7.Do you have a say on completion  deadlines?............................................................. | ❒ | ❒ | ❒ | ❒ |
| 8.Can you decide for yourself how much time  you spend on a particular task?............................ | ❒ | ❒ | ❒ | ❒ |
| 9.Do you solve day-to-day work problems  yourself?.............................................................. | ❒ | ❒ | ❒ | ❒ |
| 10.Can you plan your own work?....................... | ❒ | ❒ | ❒ | ❒ |
| 11.Can you determine for yourself the content of your work?.......................................................... | ❒ | ❒ | ❒ | ❒ |

| **Skill discretion** | **never** | **sometimes** | **often** | **always** |
| --- | --- | --- | --- | --- |
| 1.Do you have to do the same things time and  time again?......................................................... | ❒ | ❒ | ❒ | ❒ |
| 2.Does your work require creativity?................. | ❒ | ❒ | ❒ | ❒ |
| 3.Is your work varied?........................................ | ❒ | ❒ | ❒ | ❒ |
| 4.Does your work call for your own input?........ | ❒ | ❒ | ❒ | ❒ |
| 5.Does your work make sufficient demands on  all your skills and abilities?................................ | ❒ | ❒ | ❒ | ❒ |
| 6.Do you have enough variation in your  work?................................................................. | ❒ | ❒ | ❒ | ❒ |

**Indicate how true the following statements are for your current job? You may choose between never, sometimes, often or always**

| **Work demands** | **never** | **sometimes** | **often** | **always** |
| --- | --- | --- | --- | --- |
| 1.Do you have to work very fast?........................ | ❒ | ❒ | ❒ | ❒ |
| 2.Do you have too much to do?........................... | ❒ | ❒ | ❒ | ❒ |
| 3.Do you have to work extra hard to finish  something?........................................................... | ❒ | ❒ | ❒ | ❒ |
| 4.Do you have to work against the clock?.......... | ❒ | ❒ | ❒ | ❒ |
| 5.Can you briefly interrupt your work if  needed?............................................................... | ❒ | ❒ | ❒ | ❒ |
| 6.Do you have to hurry?..................................... | ❒ | ❒ | ❒ | ❒ |
| 7.Do you have to deal with getting behind  with your work?.................................................. | ❒ | ❒ | ❒ | ❒ |
| 8.Do you have too little work to do?.................. | ❒ | ❒ | ❒ | ❒ |
| 9.Do you have problems with the pace of  work? …………………………………………. | ❒ | ❒ | ❒ | ❒ |
| 10.Do you have problems with the pressure of  work?................................................................... | ❒ | ❒ | ❒ | ❒ |
| 11. Would you like to work at gentler pace?...... | ❒ | ❒ | ❒ | ❒ |

| **Co-worker support** | **never** | **sometimes** | **often** | **always** |
| --- | --- | --- | --- | --- |
| 1.Can you count on your colleagues if you run into difficulties?................................................ | ❒ | ❒ | ❒ | ❒ |
| 2.Can you ask your colleagues for help if necessary?............................................................ | ❒ | ❒ | ❒ | ❒ |
| 3.Are you on good terms with your colleagues?. | ❒ | ❒ | ❒ | ❒ |
| 4.Do you have conflicts with your colleagues?... | ❒ | ❒ | ❒ | ❒ |
| 5.Do you feel respected for your work by your colleagues?......................................................... | ❒ | ❒ | ❒ | ❒ |
| 6.Do you have to deal with hostility from your colleagues?......................................................... | ❒ | ❒ | ❒ | ❒ |
| 7.Are your colleagues friendly towards you?..... | ❒ | ❒ | ❒ | ❒ |
| 8.Is there a good atmosphere between you and your colleagues ?............................................... | ❒ | ❒ | ❒ | ❒ |
| 9.Do unpleasant situations arise between you and your colleagues?................................... | ❒ | ❒ | ❒ | ❒ |

**Indicate how true the following statements are for your current job? You may**

**choose between never, sometimes, often or always**

| **Supervisor support** | **never** | **sometime** | **often** | **always** |
| --- | --- | --- | --- | --- |
| 1.Can you rely on your immediate supervisor when you experience problems in your work?.... | ❒ | ❒ | ❒ | ❒ |
| 2.Can you ask your immediate supervisor for help if necessary?................................................ | ❒ | ❒ | ❒ | ❒ |
| 3.Are you on good terms with your immediate supervisor?........................................................ | ❒ | ❒ | ❒ | ❒ |
| 4.Do you have conflicts with your immediate supervisor?........................................................ | ❒ | ❒ | ❒ | ❒ |
| 5.Do you feel respected for your work by your immediate supervisor?........................................ | ❒ | ❒ | ❒ | ❒ |
| 6.Do you have to deal with hostility from your supervisor?......................................................... | ❒ | ❒ | ❒ | ❒ |
| 7.Is there a good atmosphere between you and your immediate supervisor ?.............................. | ❒ | ❒ | ❒ | ❒ |
| 8.Do unpleasant situations arise between you and your colleagues?.................................... | ❒ | ❒ | ❒ | ❒ |

# Part 5: General Health Status

**Indicate if the following statements are true or not for your current job?**

Need for recover- scale yes no

| 1.I find it hard to relax at the end of a working day………………….. | ❒ | ❒ |
| --- | --- | --- |
| 2.At the end of a working day I am really feeling worn – out……….. | ❒ | ❒ |
| 3.My job causes me to feel rather exhausted at the end of a working day… | ❒ | ❒ |
| 4.Generally speaking, I am still feeling fresh after supper…………… | ❒ | ❒ |
| 5.Generally speaking, I am able to relax only on a second day off….. | ❒ | ❒ |
| 6.I have complaints concentrating in the hours off after my working day… | ❒ | ❒ |
| 7.I find it hard to show interest in other people when I just came home  from work………………………………………………………………….. | ❒ | ❒ |
| 8.In general, it takes me over an hour to feel fully recovered after work…. | ❒ | ❒ |
| 9.When I get home, people should leave me alone for some time…… | ❒ | ❒ |
| 10.After a working day, I am often too tired to start other activities… | ❒ | ❒ |
| 11.During the last part of the working day I cannot optimally perform  my job because of fatigue sometimes………………………………… | ❒ | ❒ |

#### Subjective health complaints- scale yes no

| 1. Do you often have pains in the chest or heart region?…………….. | ❒ | ❒ |
| --- | --- | --- |
| 2. Do you often have a squeezing or blown – up feeling in the stomach region ?……………….…………………………………………….. | ❒ | ❒ |
| 3. Are you often short of breath ?…………………………………….. | ❒ | ❒ |
| 4. Is your stomach regularly upset ?………….……………………….. | ❒ | ❒ |
| 5. Do your bones or muscles ever ache ?……………………………… | ❒ | ❒ |
| 6. Are you often troubled by back – ache ?……………………………. | ❒ | ❒ |
| 7. Do you often feel tired ?…………………………………………….. | ❒ | ❒ |
| 8. Do you often have headaches ?……………………………………... | ❒ | ❒ |
| 9. Do you often feel dizzy ?…………………………………………… | ❒ | ❒ |
| 10. Do your arms and legs often numb or tingle ?…………………….. | ❒ | ❒ |
| 11. Do you often feel listless ?………………………………………… | ❒ | ❒ |
| 12. Do you normally feel tired when you get up in the morning ?…… | ❒ | ❒ |
| 13. Do you get tired sooner than you would consider normal ?……… | ❒ | ❒ |

# Part 6: Musculoskeletal problems

# Neck

# houlders

| **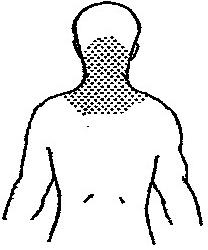** |
| --- |

**In this picture you can see the part of the body referred to in this section. Neck problems include pain, discomfort, stiffness or numbness in the shaded area, whether or not it extends from here to one or both hand/wrist.**

| 1. Have you ever had neck complaints? | ❒ yes ❒ no |
| --- | --- |

If you answered **no** to question 1, please go to **Shoulder** section

| 2. Have you **ever** been hospitalized because of neck complaints? | ❒ yes ❒ no |
| --- | --- |
| 3. Have you **ever** changed jobs because of neck complaints? | ❒ yes ❒ no |
| 4. **In the past 12 months** have you had neck complaints?  What was the diagnosis………………………….. | ❒ yes ❒ no |

If you answered **no** to question 4, please go to **Shoulder** section

5. Where your neck complaints **in the past 12 months** associated with:

| - work? | ❒ yes ❒ maybe ❒ no |
| --- | --- |
| - sports? | ❒ yes ❒ maybe ❒ no |
| - other activities in leisure time? | ❒ yes ❒ maybe ❒ no |

| 6. How long was the **longest spell** of neck complaints  **in the past 12 months?** | ❒ 1-7 days  ❒ between 2 and 3 weeks  ❒ between 3 and 4 weeks  ❒ between 2 and 3 months  ❒ longer than 3 months |
| --- | --- |
| 7. What was the **total length of time** (all spells added-  up) that you have had neck complaints **in the** **past 12**  **months?** | ❒ shorter than 4 weeks  ❒ between 2 and 3 months  ❒ between 3 and 6 month  ❒ longer than 6 months |

| 8. How **often in the past 12 months** have you had  separate spells of neck complaints? | ❒ 1 time  ❒ between 2 and 5 times  ❒ more than 5 times |
| --- | --- |
| 9. Was the onset of your neck complaints **in the past**  **12 months** sudden or gradual? | ❒ sudden  ❒ gradual |
| 10. Could you describe the nature of your neck complaints **in the past 12** **months** ?  (more than one answer is possible) | ❒ stiffness  ❒ nagging feeling  ❒ numbness  ❒ tingling  ❒ loss of strength  ❒ cramp, spasm  ❒ pain  ❒ other…………… |
| 11. Have you experienced in the past 12 months that  your neck complaints radiated to:  - left elbow?  - right elbow?  - left wrist/ hand?  - right wrist/ hand? | ❒ yes ❒ no  ❒ yes ❒ no  ❒ yes ❒ no  ❒ yes ❒ no |
| 12. How often have you been seen by an expert  because of your neck complaints **in the past 12**  **months?** | ❒ Your GP _____times  ❒ A physiotherapist ___ _times  ❒ A specialist ____ times  specify………………………..  ❒ no visit |
| 13. Which treatment(s) have you received **in the**  **past 12 months** because of your neck complaints? | ………………………………………………………………………………………………………………………………………………………………………………………………………………………………………………… |
| 14. How often have you taken sick leave **in the past**  **12 months** because of your neck complaints? | ❒ 0 times  ❒ 1 time  ❒ 2 to 5 times  ❒ over 5 times |
| 15. What is the total number of days with sick leave  **in the past 12 months** because of your neck  complaints? | ❒ 0 days  ❒ 1 to 7 days  ❒ 8 to 14 days  ❒ over 2 week |

16. In the past 12 months, how much has your neck pain changed your ability to work, where 0 is “no change” and 10 is extreme change”?

| No change | ❒  0 | ❒  1 | ❒  2 | ❒  3 | ❒  4 | ❒  5 | ❒  6 | ❒  7 | ❒  8 | ❒  9 | ❒  10 | Extreme change |
| --- | --- | --- | --- | --- | --- | --- | --- | --- | --- | --- | --- | --- |

# Shoulders

| 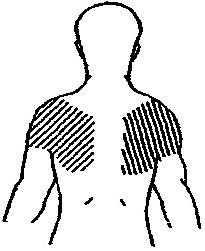 |
| --- |

**In this picture you can see the part of the body referred to in this section. Shoulder problems include pain, discomfort, stiffness or numbness in the shaded area.**

| 1. Have you ever had shoulder complaints? | ❒ yes ❒ no |
| --- | --- |

If you answered **no** to question 1, please go to **Elbows** section

| 2. Have you **ever** been hospitalized because of shoulder complaints? | ❒ yes ❒ no |
| --- | --- |
| 3. Have you **ever** changed jobs because of shoulder complaints? | ❒ yes ❒ no |
| 4. In the past 12 months have you had shoulder complaints?    What was the diagnosis………………………….. | ❒ yes ❒ no |

If you answered no to question 4, please go to **Elbows** section

5. Where your shoulder complaints **in the past 12 months** associated with:

| -in your work? | ❒ yes ❒ maybe ❒ no |
| --- | --- |
| -sports? | ❒ yes ❒ maybe ❒ no |
| -other activities in leisure time? | ❒ yes ❒ maybe ❒ no |

| 6. How long was the **longest spell** of shoulder  complaints **in the past 12 months?** | ❒ 1-7 days  ❒ between 2 and 3 weeks  ❒ between 3 and 4 weeks  ❒ between 2 and 3 months  ❒ longer than 3 months |
| --- | --- |

| 7. What was the **total length of time** (all spells  added-up) that you have had shoulder complaints  **in the past 12 months?** | ❒ shorter than 4 weeks  ❒ between 2 and 3 months  ❒ between 3 and 6 month  ❒ longer than 6 months |
| --- | --- |

| 8. How often **in the past 12 months** have you  had separate spells of shoulder complaints? | ❒ 1 time  ❒ between 2 and 5 times  ❒ more than 5 times |
| --- | --- |
| 9. Was the onset of your shoulder complaints **in**  **the past 12 months** sudden or gradual? | ❒ sudden  ❒ gradual |
| 10. Could you describe the nature of your shoulder complaints **in the past 12** **months** ?  (more than one answer is possible) | ❒ stiffness  ❒ nagging feeling  ❒ numbness  ❒ tingling  ❒ loss of strength  ❒ cramp, spasm  ❒ pain  ❒ others……………………. |
| 11. How often have you been seen by an expert  because of your shoulder complaints **in the**  **past 12 months?** | ❒ Your GP _____times  ❒ A physiotherapist _____times  ❒ A specialist ____ times  specify………………………..  ❒ no visit |
| 12. Which treatment(s) have you received **in the past 12 months** because of your shoulder complaints? | …………………………………………………………………………  ………………………………………………………………………………………………………………………………………………………………………………………… |
| 13. How often do you have taken sick leave **in the past 12 months** because of your shoulder complaints? | ❒ 0 times  ❒ 1 time  ❒ 2 to 5 times  ❒ over 5 times |
| 14. What is the total number of days with sick leave **in the past 12 months** because of your shoulder complaints? | ❒ 0 days  ❒ 1 to 7 days  ❒ 8 to 14 days  ❒ over 2 week |

15. In the past 12 months, how much has your shoulders pain changed your ability to work, where 0 is “no change” and 10 is extreme change”?

| No change | ❒  0 | ❒  1 | ❒  2 | ❒  3 | ❒  4 | ❒  5 | ❒  6 | ❒  7 | ❒  8 | ❒  9 | ❒  10 | Extreme change |
| --- | --- | --- | --- | --- | --- | --- | --- | --- | --- | --- | --- | --- |

# Elbows


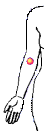


|  |
| --- |

**In this picture you can see the part of the body referred to in this section. Elbow problems include pain, discomfort, stiffness or numbness in the shaded area.**

| 1. Have you ever had elbow complaints? | ❒ yes ❒ no |
| --- | --- |

If you answered **no** to question 1, please go to **Wrist/hand** section

| 2. Have you **ever** been hospitalized because of elbow complaints? | ❒ yes ❒ no |
| --- | --- |
| 3. Have you **ever** changed jobs because of elbow complaints? | ❒ yes ❒ no |
| 4. **In the past 12 months** have you had elbow complaints?  What was the diagnosis………………………….. | ❒ yes ❒ no |

If you answered no to question 4, please go to **Wrist/hand** section

5. Where your elbow complaints **in the past 12 months** associated with:

| -work? | ❒ yes ❒ maybe ❒ no |
| --- | --- |
| -sports? | ❒ yes ❒ maybe ❒ no |
| -other activities in leisure time? | ❒ yes ❒ maybe ❒ no |

| 6. How long was the **longest spell** of elbow  complaints **in the past 12 months?** | ❒ 1-7 days  ❒ between 2 and 3 weeks  ❒ between 3 and 4 weeks  ❒ between 2 and 3 months  ❒ longer than 3 months |
| --- | --- |

| 7. What was the **total length of time** (all spells  added-up) that you have had elbow complaints  **in the past 12 months?** | ❒ shorter than 4 weeks  ❒ between 2 and 3 months  ❒ between 3 and 6 month  ❒ longer than 6 months |
| --- | --- |

| 8. How often **in the past 12 months** have you  had separate spells of elbow complaints? | ❒ 1 time  ❒ between 2 and 5 times  ❒ more than 5 times |
| --- | --- |
| 9. Was the onset of your elbow complaints **in**  **the past 12 months** sudden or gradual? | ❒ sudden  ❒ gradual |
| 10. Could you describe the nature of your elbow complaints **in the past 12** **months**?  (more than one answer is possible) | ❒ stiffness  ❒ nagging feeling  ❒ numbness  ❒ tingling  ❒ loss of strength  ❒ cramp, spasm  ❒ pain  ❒ others……………………. |
| 11. How often have you been seen by an expert  because of your elbow complaints **in the**  **past 12 months?** | ❒ Your GP _____times  ❒ A physiotherapist _____times  ❒ A specialist ____ times  specify………………………..  ❒ no visit |
| 12. Which treatment(s) have you received **in the past 12 months** because of your elbow complaints? | …………………………………………………………………………  ………………………………………………………………………………………………………………………………………………………………………………………… |
| 13. How often do you have taken sick leave **in the past 12 months** because of your elbow complaints? | ❒ 0 times  ❒ 1 time  ❒ 2 to 5 times  ❒ over 5 times |
| 14. What is the total number of days with sick leave **in the past 12 months** because of your elbow complaints? | ❒ 0 days  ❒ 1 to 7 days  ❒ 8 to 14 days  ❒ over 2 week |

15. In the past 12 months, how much has your elbows pain changed your ability to work, where 0 is “no change” and 10 is extreme change”?

| No change | ❒  0 | ❒  1 | ❒  2 | ❒  3 | ❒  4 | ❒  5 | ❒  6 | ❒  7 | ❒  8 | ❒  9 | ❒  10 | Extreme change |
| --- | --- | --- | --- | --- | --- | --- | --- | --- | --- | --- | --- | --- |

# Hand/wrist

# houlders

**
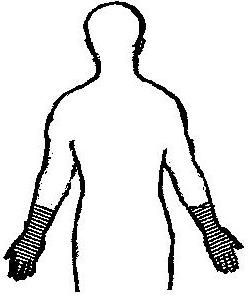
**

**In this picture you can see the part of the body referred to in this section. Hand/wrist problems include pain, discomfort, stiffness or numbness in the shaded area, independent of adjacent areas.**

| 1. Have you ever had hand/wrist complaints? | ❒ yes ❒ no |
| --- | --- |

If you answered **no** to question 1, please go to **Upper back** section

| 2. Have you **ever** been hospitalized because of hand/wrist complaints? | ❒ yes ❒ no |
| --- | --- |
| 3. Have you **ever** changed jobs because of hand/wrist complaints? | ❒ yes ❒ no |
| 4. **In the past 12 months** have you had hand/wrist complaints?  What was the diagnosis…………………… | ❒ yes ❒ no |

If you answered **no** to question 4, please go to **Upper back** section

5. Where your hand complaints **in the past 12 months** associated with:

| - work? | ❒ yes ❒ maybe ❒ no |
| --- | --- |
| - sports? | ❒ yes ❒ maybe ❒ no |
| - other activities in leisure time? | ❒ yes ❒ maybe ❒ no |

| 6. How long was the **longest spell** of hand/wrist  complaints **in the past 12 months?** | ❒ 1-7 days  ❒ between 2 and 3 weeks  ❒ between 3 and 4 weeks  ❒ between 2 and 3 months  ❒ longer than 3 months |
| --- | --- |
| 7. What was the **total length of time** (all spells added-  up) that you have had hand/wrist complaints **in** **the**  **past 12 months?** | ❒ shorter than 4 weeks  ❒ between 2 and 3 months  ❒ between 3 and 6 month  ❒ longer than 6 months |

| 8. How **often in the past 12 months** have you had  separate spells of hand/wrist complaints? | ❒ 1 time  ❒ between 2 and 5 times  ❒ more than 5 times |
| --- | --- |
| 9. Was the onset of your hand/wrist complaints **in**  **the past 12 months** sudden or gradual? | ❒ sudden  ❒ gradual |
| 10. Could you describe the nature of your hand/wrist complaints **in the past 12** **months**?  (more than one answer is possible) | ❒ stiffness  ❒ nagging feeling  ❒ numbness  ❒ tingling  ❒ loss of strength  ❒ cramp, spasm  ❒ pain  ❒ others……………………. |
| 11. How often have you been seen by an expert  because of your hand/wrist complaints **in the past**  **12 months?** | ❒ Your GP _____times  ❒ A physiotherapist _____times  ❒ A specialist _____times  specify……………...……..  ❒ no visit |
| 12. Which treatment(s) have you received **in the**  **past 12 months** because of your hand/wrist  complaints? | ………………………………………………………………………………………………………………………………………………………………………………………………………………………………………………… |
| 13. How often have you taken sick leave **in the past**  **12 months** because of your hand/wrist complaints? | ❒ 0 times  ❒ 1 time  ❒ 2 to 5 times  ❒ over 5 times |
| 14. What is the total number of days with sick leave  **in the past 12 months** because of your hand/wrist  complaints? | ❒ 0 days  ❒ 1 to 7 days  ❒ 8 to 14 days  ❒ over 2 week |

15. In the past 12 months, how much has your hands/wrists pain changed your ability to work, where 0 is “no change” and 10 is extreme change”?

| No change | ❒  0 | ❒  1 | ❒  2 | ❒  3 | ❒  4 | ❒  5 | ❒  6 | ❒  7 | ❒  8 | ❒  9 | ❒  10 | Extreme change |
| --- | --- | --- | --- | --- | --- | --- | --- | --- | --- | --- | --- | --- |

# Upper back


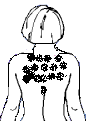


|  |
| --- |

In this picture you can see the part of the body referred to in this section. Upper back problems include pain, discomfort, or stiffness, in the shaded area, independent of adjacent areas.

| 1. Have you ever had upper back complaints? | ❒ yes ❒ no |
| --- | --- |

If you answered **no** to question 1, please go to **Lower back** section

| 2. Have you ever been hospitalized because of upper back complaints? | ❒ yes ❒ no |
| --- | --- |
| 3. Have you **ever** changed jobs because of upper back complaints? | ❒ yes ❒ no |
| 4. **In the past 12 months** have you had upper back complaints?  What was the diagnosis………………………………... | ❒ yes ❒ no |

If you answered **no** to question 4, please continue with **Lower back** section

5. Where your back complaints **in the past 12 months** associated with :

| - work ? | ❒ yes ❒ maybe ❒ no |
| --- | --- |
| - sports ? | ❒ yes ❒ maybe ❒ no |
| - other activities in leisure time ? | ❒ yes ❒ maybe ❒ no |

| 6. How long was the **longest spell** of upper back  complaints **in the past 12 months ?** | ❒ 1-7 days  ❒ between 2 and 3 weeks  ❒ between 3 and 4 weeks  ❒ between 2 and 3 months  ❒ longer than 3 months |
| --- | --- |
| 7. What was the **total length of time** (all spells added-  up) that you have had upper back complaints **in the**  **past 12 months** ? | ❒ shorter than 4 weeks  ❒ between 2 and 3 months  ❒ between 3 and 6 month  ❒ longer than 6 months |
| 8. How often in the past 12 months have you had  separate spells of upper back complaints? | ❒ 1 time  ❒ between 2 and 5 times  ❒ more than 5 times |
| 9. Was the onset of your upper back complaints  in the past 12 months sudden or gradual? | ❒ sudden  ❒ gradual |
| 10. Could you describe the nature of your upper back complaints **in the past 12** **months** ?  (more than one answer is possible) | ❒ stiffness  ❒ nagging feeling  ❒ numbness  ❒ tingling  ❒ loss of strength  ❒ cramp, spasm  ❒ pain  ❒ others….. |
| 11. How often have you been seen by an expert  because of your upper back complaints **in the**  **past 12 months** ? | ❒ Your GP ____times  ❒ A physiotherapist _times  ❒ A specialist __ _ times  specify…………………..  ❒ no visit |
| 12. Which treatment(s) have you received in the  past 12 months because of your upper back  complaints? | …………………………………………………………………………………………………………………………………………………………………………………………………………… |
| 13. How often do you have taken sick leave **in**  **the past 12 months** because of your upper back  complaints? | ❒ 0 times  ❒ 1 time  ❒ 2 to 5 times  ❒ over 5 times |
| 14. What is the total number of days with sick  leave **in the past 12 months** because of your  upper back complaints? | ❒ 0 days  ❒ 1 to 7 days  ❒ 8 to 14 days  ❒ over 2 weeks |

15. In the past 12 months, how much has your upper back pain changed your ability to work, where 0 is “no change” and 10 is extreme change”?

| No change | ❒  0 | ❒  1 | ❒  2 | ❒  3 | ❒  4 | ❒  5 | ❒  6 | ❒  7 | ❒  8 | ❒  9 | ❒  10 | Extreme change |
| --- | --- | --- | --- | --- | --- | --- | --- | --- | --- | --- | --- | --- |

# Low back

| 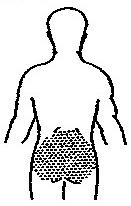 |
| --- |

In this picture you can see the part of the body referred to in this section. Low back problems include pain, discomfort, or stiffness, in the shaded area, whether or not it extends from here to one or both legs

| 1. Have you ever had low back complaints? | ❒ yes ❒ no |
| --- | --- |

If you answered **no** to question 1, please go to **Hip/thigh** section

| 2. Have you **ever** been hospitalized because of back complaints? | ❒ yes ❒ no |
| --- | --- |
| 3. Have you **ever** changed jobs because of back complaints? | ❒ yes ❒ no |
| 4. **In the past 12 months** have you had low back complaints?  What was the diagnosis…………………….. | ❒ yes ❒ no |

If you answered **no** to question 4, please continue with **Hip/thigh** section

5. Where your lower back complaints **in the past 12 months** associated with:

| - work ? | ❒ yes ❒ maybe ❒ no |
| --- | --- |
| - sports ? | ❒ yes ❒ maybe ❒ no |
| - other activities in leisure time ? | ❒ yes ❒ maybe ❒ no |
| - pregnancy (only to answer by female)? | ❒ yes ❒ maybe ❒ no |
| - menstrual period (only to answer by female)? | ❒ yes ❒ maybe ❒ no |

| 6. How long was the **longest spell** of back  complaints **in the past 12 months ?** | ❒ 1-7 days  ❒ between 2 and 3 weeks  ❒ between 3 and 4 weeks  ❒ between 2 and 3 months  ❒ longer than 3 months |
| --- | --- |
| 7. What was the **total length of time** (all spells  added-up) that you have had low back  complaints **in the past 12 months** ? | ❒ shorter than 4 weeks  ❒ between 2 and 3 months  ❒ between 3 and 6 month  ❒ longer than 6 months |
| 8. How often in the past 12 months have you  had separate spells of back complaints? | ❒ 1 time  ❒ between 2 and 5 times  ❒ more than 5 times |
| 9. Was the onset of your back complaints in the  past 12 months sudden or gradual? | ❒ sudden  ❒ gradual |
| 10. Could you describe the nature of your back  complaints **in the past 12** **months** ?  (more than one answer is possible) | ❒ stiffness  ❒ nagging feeling  ❒ numbness  ❒ tingling  ❒ loss of strength  ❒ cramp, spasm  ❒ pain  ❒ others…………… |
| 11. Have you experienced in the past 12  months back complaints radiating to   - left knee? - right knee ? - left ankle/foot? - right ankle/foot ? | ❒ yes ❒ no  ❒ yes ❒ no  ❒ yes ❒ no  ❒ yes ❒ no |
| 12. How often have you been seen by  an expert because of your back  complaints **in the past 12 months** ? | ❒ Your GP _____times  ❒ A physiotherapist _____times  ❒ A specialist ____ times  specify………………………..  ❒ no visit |
| 13. Which treatment(s) have you received in the  past 12 months because of your back  complaints? | …………………………………  …………………………………  …………………………………  ………………………………….  ……………………………………………………………………………………………………… |
| 14. How often do you have taken sick leave **in**  **the past 12 months** because of your back  complaints? | ❒ 0 times  ❒ 1 time  ❒ 2 to 5 times  ❒ over 5 times |
| 15. What is the total number of days with sick  leave **in the past 12 months** because of your  back complaints? | ❒ 0 days  ❒ 1 to 7 days  ❒ 8 to 14 days  ❒ over 2 weeks |

On the next seven questions we would like to know how serious you rate your back pain and whether your back pain affected your regular daily activities. You are asked to indicate your opinion on a scale from 0 ‘‘no problems’’ to 10 ‘‘problems as serious as possible’’

1. How would you rate your back pain at the present time on a 0-10 scale, where 0 is ‘‘no pain’’ and 10 is ‘‘pain as bad as possible’’

| No pain | ❒  0 | ❒  1 | ❒  2 | ❒  3 | ❒  4 | ❒  5 | ❒  6 | ❒  7 | ❒  8 | ❒  9 | ❒  10 | Pain as bad could be |
| --- | --- | --- | --- | --- | --- | --- | --- | --- | --- | --- | --- | --- |

#####

1. In the past 12 months, how intense was your worst back pain rated on a 0-10 scale, where 0 is ‘‘no pain’’ and 10 ‘‘pain as bad possible’’?

| No pain | ❒  0 | ❒  1 | ❒  2 | ❒  3 | ❒  4 | ❒  5 | ❒  6 | ❒  7 | ❒  8 | ❒  9 | ❒  10 | Pain as bad could be |
| --- | --- | --- | --- | --- | --- | --- | --- | --- | --- | --- | --- | --- |

1. In the past 12 months, on the average, how intense was your back pain rated on a 0-10 scale, where 0 is ‘‘no pain’’ and 10 ‘‘pain as bad possible’’?

| No pain | ❒  0 | ❒  1 | ❒  2 | ❒  3 | ❒  4 | ❒  5 | ❒  6 | ❒  7 | ❒  8 | ❒  9 | ❒  10 | Pain as bad could be |
| --- | --- | --- | --- | --- | --- | --- | --- | --- | --- | --- | --- | --- |

4. How many days **in the past 12** **months** have you been kept from your usual activities because of back pain. (work, school, house work) ?

days

5. In the **past 12 months**, how much has your back pain interfered with your daily activities (work, school, housework) rated on 0-10 scale, where 0 is “no interference” and 10 is unable to carry on any activities”?

| No interference | ❒  0 | ❒  1 | ❒  2 | ❒  3 | ❒  4 | ❒  5 | ❒  6 | ❒  7 | ❒  8 | ❒  9 | ❒  10 | Unable to carry on any activities |
| --- | --- | --- | --- | --- | --- | --- | --- | --- | --- | --- | --- | --- |

6. In the past 12 months, how much has your back pain changed your ability to take part in recreational, social and family activities, where 0 is “no change” and 10 is extreme change”?

| No change | ❒  0 | ❒  1 | ❒  2 | ❒  3 | ❒  4 | ❒  5 | ❒  6 | ❒  7 | ❒  8 | ❒  9 | ❒  10 | Extreme change |
| --- | --- | --- | --- | --- | --- | --- | --- | --- | --- | --- | --- | --- |

7. In the past 12 months, how much has your back pain changed your ability to work, where 0 is “no change” and 10 is extreme change”?

| No change | ❒  0 | ❒  1 | ❒  2 | ❒  3 | ❒  4 | ❒  5 | ❒  6 | ❒  7 | ❒  8 | ❒  9 | ❒  10 | Extreme change |
| --- | --- | --- | --- | --- | --- | --- | --- | --- | --- | --- | --- | --- |

# Hip/thigh


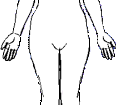


**In this picture you can see the part of the body referred to in this section. Hip/thigh problems include pain, discomfort, stiffness or numbness in the shaded area, independent of adjacent areas.**

| 1. Have you ever had hip/thigh complaints? | ❒ yes ❒ no |
| --- | --- |

If you answered **no** to question 1, please go to **Knees section**

| 2. Have you **ever** been hospitalized because of hip/thigh complaints? | ❒ yes ❒ no |
| --- | --- |
| 3. Have you **ever** changed jobs because of hip/thigh complaints? | ❒ yes ❒ no |
| 4. **In the past 12 months** have you had hip/thigh complaints?    What was the diagnosis……………………………. | ❒ yes ❒ no |

If you answered **no** to question 4, please go to **Knees section**

5. Where your hip/thigh complaints **in the past 12 months** associated with:

| - work? | ❒ yes ❒ maybe ❒ no |
| --- | --- |
| - sports? | ❒ yes ❒ maybe ❒ no |
| - other activities in leisure time? | ❒ yes ❒ maybe ❒ no |

| 6. How long was the **longest spell** of hip/thigh complaints  **in the past 12 months?** | ❒ 1-7 days  ❒ between 2 and 3 weeks  ❒ between 3 and 4 weeks  ❒ between 2 and 3 months  ❒ longer than 3 months |
| --- | --- |
| 7. What was the **total length of time** (all spells added-up)  that you have had hip/thigh complaints **in the** **past 12**  **months?** | ❒ shorter than 4 weeks  ❒ between 2 and 3 months  ❒ between 3 and 6 month  ❒ longer than 6 months |

| 8. How **often in the past 12 months** have you had  separate spells of hip/thigh complaints? | ❒ 1 time  ❒ between 2 and 5 times  ❒ more than 5 times |
| --- | --- |
| 9. Was the onset of your hip/thigh complaints in the  past 12 months sudden or gradual? | ❒ sudden  ❒ gradual |
| 10. Could you describe the nature of your hip/thigh  complaints **in the past 12** **months** ?  (more than one answer is possible) | ❒ stiffness  ❒ nagging feeling  ❒ numbness  ❒ tingling  ❒ loss of strength  ❒ cramp, spasm  ❒ pain  ❒ others…………… |
| 11. How often have you been seen by an expert because of your hip/thigh complaints **in the past 12** **months?** | ❒ Your GP ___times  ❒A physiotherapist ___times  ❒ A specialist ____times  specify…………...………...  ❒ no visit |
| 12. Which treatment(s) have you received **in the past**  **12 months** because of your hip/thigh complaints? | ……………………………………………………………………………………………………………………………………………………………………………………………………………………………… |
| 13. How often have you taken sick leave **in the past**  **12 months** because of your hip/thigh complaints? | ❒ 0 times  ❒ 1 time  ❒ 2 to 5 times  ❒ over 5 times |
| 14. What is the total number of days with sick leave **in**  **the past 12 months** because of your hip/thigh  complaints? | ❒ 0 days  ❒ 1 to 7 days  ❒ 8 to 14 days  ❒ over 2 week |

15. In the past 12 months, how much has your hip/thigh pain changed your ability to work, where 0 is “no change” and 10 is extreme change”?

| No change | ❒  0 | ❒  1 | ❒  2 | ❒  3 | ❒  4 | ❒  5 | ❒  6 | ❒  7 | ❒  8 | ❒  9 | ❒  10 | Extreme change |
| --- | --- | --- | --- | --- | --- | --- | --- | --- | --- | --- | --- | --- |

# Knees

# houlders


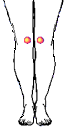


**In this picture you can see the part of the body referred to in this section. Knee problems include pain, discomfort, stiffness or numbness in the shaded area, independent of adjacent areas.**

| 1. Have you ever had knee complaints? | ❒ yes ❒ no |
| --- | --- |

If you answered **no** to question 1, please go to **Ankle/feet section**

| 2. Have you **ever** been hospitalized because of knee complaints? | ❒ yes ❒ no |
| --- | --- |
| 3. Have you **ever** changed jobs because of knee complaints? | ❒ yes ❒ no |
| 4. **In the past 12 months** have you had knee complaints?  What was the diagnosis…………………………… | ❒ yes ❒ no |

If you answered **no** to question 4, please go to **Ankle/feet section**

5. Where your knee complaints **in the past 12 months** associated with:

| - work? | ❒ yes ❒ maybe ❒ no |
| --- | --- |
| - sports? | ❒ yes ❒ maybe ❒ no |
| - other activities in leisure time? | ❒ yes ❒ maybe ❒ no |

| 6. How long was the **longest spell** of knee complaints **in**  **the past 12 months?** | ❒ 1-7 days  ❒ between 2 and 3 weeks  ❒ between 3 and 4 weeks  ❒ between 2 and 3 months  ❒ longer than 3 months |
| --- | --- |
| 7. What was the **total length of time** (all spells added-  up) that you have had knee complaints **in the** **past 12**  **months?** | ❒ shorter than 4 weeks  ❒ between 2 and 3 months  ❒ between 3 and 6 month  ❒ longer than 6 months |

| 8. How **often in the past 12 months** have you had  separate spells of knee complaints? | ❒ 1 time  ❒ between 2 and 5 times  ❒ more than 5 times |
| --- | --- |
| 9. Was the onset of your knee complaints in the  past 12 months sudden or gradual? | ❒ sudden  ❒ gradual |
| 10. Could you describe the nature of your knee  complaints **in the past 12** **months** ?  (more than one answer is possible) | ❒ stiffness  ❒ nagging feeling  ❒ numbness  ❒ tingling  ❒ loss of strength  ❒ cramp, spasm  ❒ pain  ❒ others…………… |
| 11. How often have you been seen by an expert  because of your knee complaints **in the past 12**  **months?** | ❒ Your GP ___times  ❒ A physiotherapist ___times  ❒ A specialist ____times  specify………………...…..  ❒ no visit |
| 12. Which treatment(s) have you received **in the past**  **12 months** because of your knee complaints? | ……………………………………………………………………………………………………………………………………………………………………………………………………………………………… |
| 13. How often have you taken sick leave **in the past**  **12 months** because of your knee complaints? | ❒ 0 times  ❒ 1 time  ❒ 2 to 5 times  ❒ over 5 times |
| 14. What is the total number of days with sick leave **in**  **the past 12 months** because of your knee complaints? | ❒ 0 days  ❒ 1 to 7 days  ❒ 8 to 14 days  ❒ over 2 week |

15. In the past 12 months, how much has your knee pain changed your ability to work, where 0 is “no change” and 10 is extreme change”?

| No change | ❒  0 | ❒  1 | ❒  2 | ❒  3 | ❒  4 | ❒  5 | ❒  6 | ❒  7 | ❒  8 | ❒  9 | ❒  10 | Extreme change |
| --- | --- | --- | --- | --- | --- | --- | --- | --- | --- | --- | --- | --- |

# Ankle/feet

# houlders


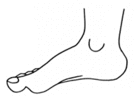


**In this picture you can see the part of the body referred to in this section. Ankle/feet problems include pain, discomfort, stiffness or numbness in the shaded area, independent of adjacent areas.**

| 1. Have you ever had ankle/feet complaints? | ❒ yes ❒ no |
| --- | --- |

If you answered **no** to question 1, please do not continue

| 2. Have you **ever** been hospitalized because of ankle/feet complaints? | ❒ yes ❒ no |
| --- | --- |
| 3. Have you **ever** changed jobs because of ankle/feet complaints? | ❒ yes ❒ no |
| 4. **In the past 12 months** have you had ankle/feet complaints?    What was the diagnosis……………………….. | ❒ yes ❒ no |

If you answered **no** to question 4, please do not continue

5. Where your ankle/feet complaints **in the past 12 months** associated with:

| - work? | ❒ yes ❒ maybe ❒ no |
| --- | --- |
| - sports? | ❒ yes ❒ maybe ❒ no |
| - other activities in leisure time? | ❒ yes ❒ maybe ❒ no |

| 6. How long was the **longest spell** of ankle/feet  complaints **in the past 12 months?** | ❒ 1-7 days  ❒ between 2 and 3 weeks  ❒ between 3 and 4 weeks  ❒ between 2 and 3 months  ❒ longer than 3 months |
| --- | --- |
| 7. What was the **total length of time** (all spells  added-up) that you have had ankle/feet complaints **in**  **the past 12 months?** | ❒ shorter than 4 weeks  ❒ between 2 and 3 months  ❒ between 3 and 6 month  ❒ longer than 6 months |

| 8. How **often in the past 12 months** have you had  separate spells of ankle/feet complaints? | ❒ 1 time  ❒ between 2 and 5 times  ❒ more than 5 times |
| --- | --- |
| 9. Was the onset of your ankle/feet complaints in the  past 12 months sudden or gradual? | ❒ sudden  ❒ gradual |
| 10. Could you describe the nature of your ankle/feet  complaints **in the past 12** **months** ?  (more than one answer is possible) | ❒ stiffness  ❒ nagging feeling  ❒ numbness  ❒ tingling  ❒ loss of strength  ❒ cramp, spasm  ❒ pain  ❒ others…………… |
| 11. How often have you been seen by an expert  because of your ankle/feet complaints **in the past 12**  **months?** | ❒ Your GP ___times  ❒ A physiotherapist ___times  ❒ A specialist ____times  specify………………...…..  ❒ no visit |
| 12. Which treatment(s) have you received **in the past**  **12 months** because of your ankle/feet complaints? | ……………………………………………………………………………………………………………………………………………………………………………………………………………………………… |
| 13. How often have you taken sick leave **in the past**  **12 months** because of your ankle/feet complaints? | ❒ 0 times  ❒ 1 time  ❒ 2 to 5 times  ❒ over 5 times |
| 14. What is the total number of days with sick leave **in**  **the past 12 months** because of your ankle/feet  complaints? | ❒ 0 days  ❒ 1 to 7 days  ❒ 8 to 14 days  ❒ over 2 week |

15. In the past 12 months, how much has your ankle/feet pain changed your ability to work, where 0 is “no change” and 10 is extreme change”?

| No change | ❒  0 | ❒  1 | ❒  2 | ❒  3 | ❒  4 | ❒  5 | ❒  6 | ❒  7 | ❒  8 | ❒  9 | ❒  10 | Extreme change |
| --- | --- | --- | --- | --- | --- | --- | --- | --- | --- | --- | --- | --- |
